# Desflurane Attenuates Ventilator-Induced Lung Injury in Rats with Acute Respiratory Distress Syndrome

**DOI:** 10.1155/2018/7507314

**Published:** 2018-01-29

**Authors:** Xue Lin, Ying-nan Ju, Wei Gao, Dong-mei Li, Chang-chun Guo

**Affiliations:** ^1^Department of Anesthesiology, The Second Affiliated Hospital of Harbin Medical University, Harbin, Heilongjiang Province 150081, China; ^2^Department of Intensive Care Unit, Harbin Medical University Cancer Hospital, Harbin, Heilongjiang Province 150081, China

## Abstract

Ventilator-induced lung injury aggravates the existing lung injury. This study investigated the effect of desflurane on VILI in a rat model of acute respiratory distress syndrome. Forty-eight rats were randomized into a sham (S) group, control (C) group, lipopolysaccharide/ventilation (LV) group, lipopolysaccharide/ventilation/desflurane (LVD) group, or lipopolysaccharide/low ventilation with and without desflurane (LLV and LLVD) groups. Rats in the S group received anesthesia only. Rats in the LV and LVD groups received lipopolysaccharide and were ventilated with a high tidal volume. Rats in LLV and LLVD groups were treated as the LV and LVD groups and ventilated with a low tidal volume. PaO_2_/FiO_2_, lung wet-to-dry weight ratios, concentrations of inflammatory factors in serum and BALF, histopathologic analysis of lung tissue, and levels of nuclear factor- (NF-) *κ*B protein in lung tissue were investigated. PaO_2_/FiO_2_ was significantly increased by desflurane. Total cell count, macrophages, and neutrophils in BALF and proinflammatory factors in BALF and serum were significantly decreased by desflurane, while IL-10 was increased. The histopathological changes and levels of NF-*κ*B protein in lung tissue were decreased by desflurane. The results indicated that desflurane ameliorated VILI in a rat model of acute respiratory distress syndrome.

## 1. Background

Mechanical ventilation is an essential part of life support in the intensive care unit, but it can lead to ventilator-induced lung injury (VILI) [1, 2]. An estimated 24% of all patients that are mechanically ventilated develop VILI [3], and patients with acute respiratory distress syndrome (ARDS) are particularly susceptible to VILI. Notably, these patients frequently require increased airway pressure and large volume mechanical ventilation to maintain oxygenation. Although lung protective strategies may help to relieve lung injury [4], VILI remains a major complication associated with the long-term use of mechanical ventilation [5, 6].

Evidence suggests that the NF-*κ*B-mediated inflammatory response plays a pivotal role in the development of ARDS and VILI [7, 8]. Therefore, effectively targeting the expression of NF-*κ*B is a potential strategy for prevention of VILI.

Volatile anesthetics may protect against lung injury [9, 10]. Desflurane is a widely used volatile anesthetic that has anti-inflammatory properties [11, 12]. Studies show that desflurane can decrease shunt fraction and lung perfusion of the nonindependent lung during one-lung ventilation, [13] reduce basal airway tone in acetylcholine-induced bronchoconstriction [14], and decrease the inflammatory response by interfering with CXC receptor-2 signaling in isolated human neutrophils [15]. The low blood/gas partition coefficient of desflurane allows for rapid induction/recovery, which is especially useful during lengthy surgeries. However, some reports suggest that desflurane induces airway constriction, increases lung microvascular permeability, and aggravates lung injury [16–18], and data describing the contribution of desflurane to VILI in patients with ARDS are scarce. To address these disparate findings, the present study was performed to test the effect of desflurane on VILI in an ARDS model and investigate whether desflurane is suitable for use in patients with lung dysfunction. ARDS was induced in rats with a sublethal dose of lipopolysaccharide. Data from this study may inform clinical decisions for general anesthesia in mechanically ventilated patients with prior lung injury.

## 2. Materials and Methods

### 2.1. Study Design

The study design and methodology are as previously published [19]. Briefly, adult (250–300 g) male Wistar rats (*n* = 64) were purchased from the Second Affiliated Hospital of Harbin Medical University, Harbin, China, and were treated according to national guidelines. All experiments were performed in accordance with Harbin Medical University's Institutional Animal Care and Use Committee. Rats were fasted for 24 hours before the study, but water was provided ad libitum.

Rats were randomized into 6 groups (*n* = 8 each): sham (S), control (C), lipopolysaccharide/ventilation (LV), lipopolysaccharide/ventilation/desflurane (LVD), or lipopolysaccharide/low ventilation with and without desflurane (LLV and LLVD). All rats were anesthetized with pentobarbital sodium 30 mg/kg and rocuronium 0.6 mg/kg via intraperitoneal (i.p.) injection. Anesthesia was maintained with 3% pentobarbital sodium (10 mg/kg) and rocuronium (0.6 mg/kg) for a 1-hour interval.

Anesthesia was maintained with 3% pentobarbital sodium (10 mg/kg), and muscle relaxation was maintained with intermittent injections of rocuronium (0.6 mg/kg) for 4.5 hours. The caudal vein and artery of rats were canulated. Rats in the S group received anesthesia only. Rats in LV and LVD groups received lipopolysaccharide 500 *μ*g/kg (*Escherichia coli* lipopolysaccharide, 0111:B4, Sigma, Saint Louis, Missouri, USA) by intravenous injection and were intubated. After 30 mins, rats in the LV group were mechanically ventilated with 80% oxygen and 20% nitrogen for 4 hours tidal volume 30 ml/kg [20] (inspiratory expiratory ratio: 1 : 1, respiratory rate: 50/min, FiO_2_: 80%), while rats in the LVD group received 80% oxygen, 12% desflurane, and 8% nitrogen. Rats in LLV and LLVD groups received the same dosage of lipopolysaccharide as rats in the LV and LVD groups but were ventilated with a low tidal volume (10 ml/kg) for 4 hours. Rats in the C group were mechanically ventilated (tidal volume 30 ml/kg) with 80% oxygen and 20% nitrogen for 4 hours without lipopolysaccharide. During anesthesia and ventilation, the temperature of all rats was monitored and maintained between 35°C and 37°C using a thermal blanket.

### 2.2. Tissue Analyses

#### 2.2.1. Arterial Blood Gases

Arterial blood gases were analyzed at baseline, 30 minutes after lipopolysaccharide injection, and after mechanical ventilation using the Bayer Rapidlab 348 (Bayer Diagnostics, Germany).

#### 2.2.2. BALF and Serum

Peripheral blood was sampled at baseline, 30 minutes after lipopolysaccharide injection, and after mechanical ventilation.

Bronchoalveolar lavage fluid (BALF) was prepared from the left lung by injecting and withdrawing saline (15 ml/kg). BALF was centrifuged at 1,000 g for 15 minutes at 4°C and stored at −80°C until analysis. The Bradford method was used to measure total protein in BALF. The number of, macrophages, neutrophils, and total cells in BALF were measured with a cell counter.

Tumor necrosis factor- (TNF-) *α*, interleukin- (IL-) 1*β*, IL-8, IL-10, and neutrophil elastase concentrations were measured in serum and BALF using commercially available ELISA kits according to the manufacturer's instructions (Wuhan Boster Bio-Engineering Limited Company, Wuhan, Hubei, China).

#### 2.2.3. Pulmonary Alveolocapillary Permeability

Pulmonary alveolocapillary permeability was measured by calculating the wet/dry weight ratio of a portion of the right lung from sacrificed rats.

### 2.3. Histopathologic Analysis

A portion of the right lung from sacrificed rats was fixed with 10% formalin and embedded in paraffin. Four *μ*m sections were cut and stained with hematoxylin and eosin. Lung injury was evaluated by an independent pathologist who was blinded to the grouping under light microscopy, taking into account hemorrhage in the lung tissue, alveolar congestion, edema, infiltration of macrophages and neutrophils, and morphological changes in the alveolar wall. Lung injury was quantified by two independent pathologists who did not participate in the study. The pathologists analyzed lung histopathology and scored lung injury on a scale from 0 to 4, where 0 represented minimum damage; 1 represented mild damage; 2 represented moderate damage; 3 represented severe damage; and 4 represented maximum damage.

### 2.4. Western Blot Analysis

Protein was extracted from a portion of the right lung tissue of sacrificed rats and homogenized. Aliquots of protein homogenate were separated on polyacrylamide gels and transferred onto polyvinylidene fluoride membranes. Membranes were blocked with 5% milk powder, probed with primary antibodies against NF-*κ*B p50, and incubated with horseradish peroxidase-linked secondary antibodies (Santa Cruz Biotechnology, Santa Cruz, CA, USA). Bands were visualized using enhanced chemiluminescence.

### 2.5. Statistical Analysis

Data were analyzed using SPSS version 11.0 (SPSS, Chicago, IL, USA). Data are presented as means ± SD. All data obtained during the mechanical ventilation period were included in the analyses. Single data points were compared using the ANOVA with a post hoc Bonferroni correction. Survival curves were derived using the Kaplan-Meier method, and differences were evaluated with the log-rank test. *P* < 0.05 was considered statistically significant.

## 3. Investigations and Results

### 3.1. Desflurane Improves Alveolocapillary Permeability in VILI

**Figure 1:**
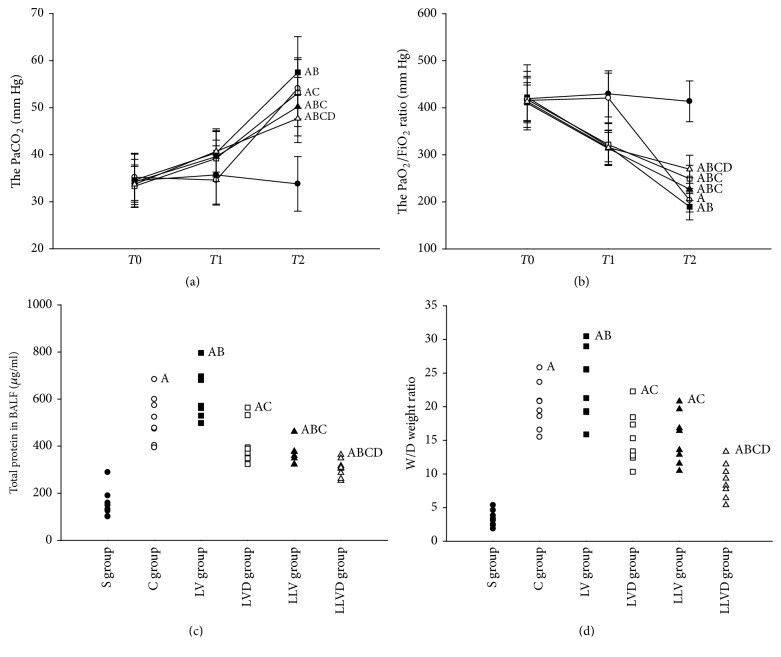
PaO_2_/FiO_2_ ratio, PaCO_2_, wet/dry weight ratio, and protein concentrations. ^A^*P* < 0.05 versus S group; ^B^*P* < 0.05 versus C group; ^C^*P* < 0.05 versus LV group; ^D^*P* < 0.05 versus LLV group. ●, S group; ○, C group; ■, LV group; □, LVD group; ▲, LLV group; △, LLVD group). (*T*0, baseline; *T*1, 30 minutes after lipopolysaccharide injection; and *T*2, after mechanical ventilation.

Compared to rats in the S group, arterial oxygen partial pressure/fractional inspired oxygen (PaO_2_/FiO_2_) was significantly decreased in rats in the LV, LVD, LLV, and LLVD groups (*P* < 0.05). Compared to the LV and LLV group, PaO_2_/FiO_2_ was significantly increased in the LVD and LLVD groups, respectively (*P* < 0.05). Compared to the LVD group, PaO_2_/FiO_2_ was increased in the LLVD group, but the difference was not significant. In contrast to the PaO_2_/FiO_2_ ratio, PaCO_2_ in the LV and LVD groups was significantly increased compared to the S group. PaCO_2_ in the LLV and LLVD groups were higher than in the S group, but the difference was not significant (Figure 1). Total protein and the wet/dry weight ratio in BALF were significantly decreased in the LVD group compared to the LV group (Figure 1). Similarly, total protein and wet/dry weight ratio were significantly decreased in the LLVD group compared to the LLV group (*P* < 0.05) (Figure 1).

### 3.2. Desflurane Inhibits Inflammation in VILI

**Figure 2:**
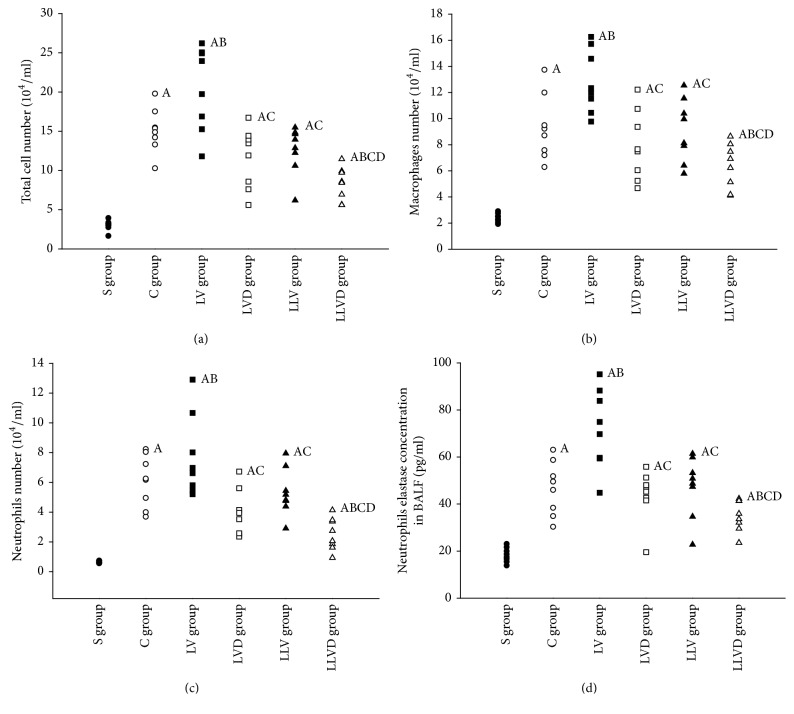
Total cell count, macrophages, neutrophil, and neutrophil elastase levels in BALF. ^A^*P* < 0.05 versus S group; ^B^*P* < 0.05 versus C group; ^C^*P* < 0.05 versus LV group; ^D^*P* < 0.05 versus LLV group. ●, S group; ○, C group; ■, LV group; □, LVD group; ▲, LLV group; and △, LLVD group.

**Figure 3:**
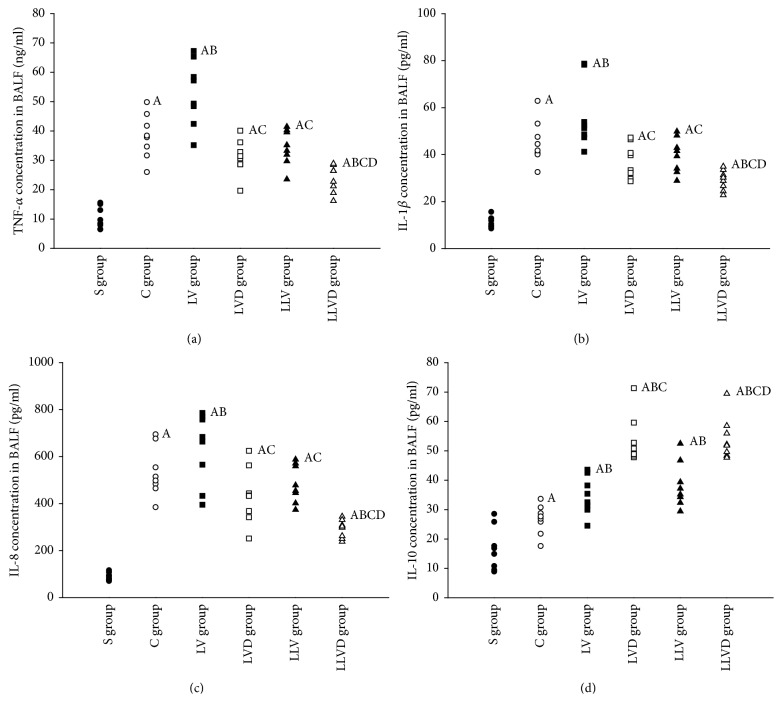
TNF-*α*, IL-1*β*, IL-8, and IL-10 levels in BALF. ^A^*P* < 0.05 versus S group; ^B^*P* < 0.05 versus C group; ^C^*P* < 0.05 versus LV group; ^D^*P* < 0.05 versus LLV group. ●, S group; ○, C group; ■, LV group; □, LVD group; ▲, LLV group; and △, LLVD group.

**Figure 4:**
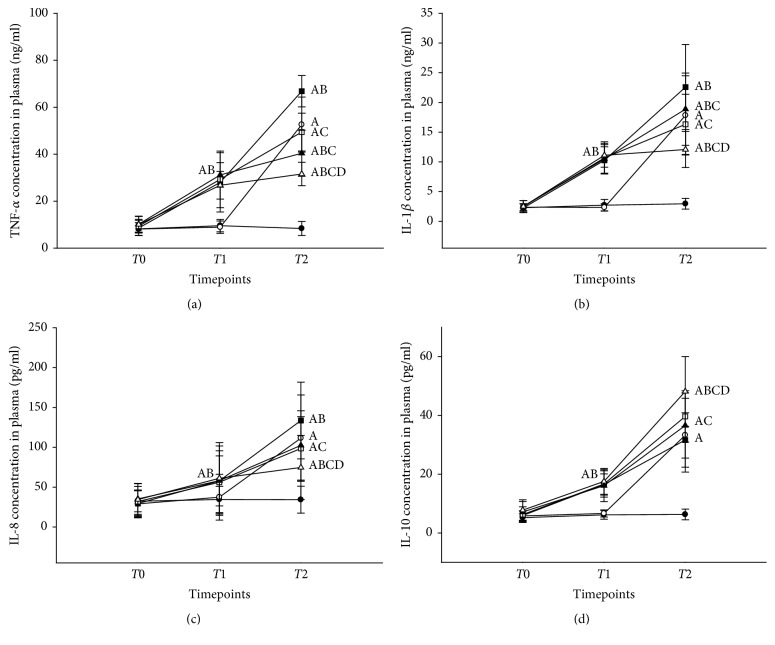
TNF-*α*, IL-1*β*, IL-8, and IL-10 levels in serum. ^A^*P* < 0.05, versus S group; ^B^*P* < 0.05, versus C group; ^C^*P* < 0.05, versus LV group; ^D^*P* < 0.05 versus LLV group. ●, S group; ○, C group; ■, LV group; □, LVD group; ▲, LLV group; and △, LLVD group.

Compared to rats in the S group, total cells, macrophages, and neutrophils in BALF were significantly increased in rats in the C, LV, LVD, LLV, and LLVD groups (Figure 2). Total cell count, macrophages, neutrophils, and the concentration of neutrophil elastase in BALF were decreased in the LVD group compared to the LV group (all *P* < 0.05). The inflammatory cell count and elastase concentration in the LLVD group were significantly decreased compared to the LVD group (Figure 2). Compared to rats in the S group, the concentrations of the proinflammatory factors TNF-*α*, IL-1*β*, and IL-8 in serum and BALF were higher in rats in the C, LV, and LVD groups (all *P* < 0.05). The concentrations of the proinflammatory factors TNF-*α*, IL-1*β*, and IL-8 in serum and BALF were significantly decreased, and the concentration of the anti-inflammatory factor IL-10 was increased in the LVD group compared to the LV group (*P* < 0.05). Proinflammatory factor concentrations were significantly decreased in the LLVD group compared to the LLV group, but the concentration of the anti-inflammatory IL-10 was increased by desflurane (*P* < 0.05) (Figures 3 and 4).

**Figure 5:**
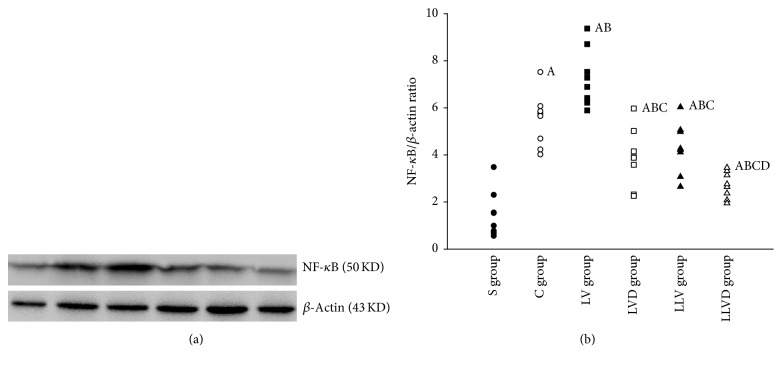
NF-*κ*B p50 levels in lung tissue. ^A^*P* < 0.05 versus S group; ^B^*P* < 0.05 versus C group; ^C^*P* < 0.05 versus LV group; ^D^*P* < 0.05 versus LLV group. ●, S group; ○, C group; ■, LV group; □, LVD group; ▲, LLV group; and △, LLVD group.

Compared to rats in the S group, after 4 hours of ventilation, the levels of NF-*κ*B protein were increased in rats in the C, LV, LVD, LLV, and LLVD groups (all *P* < 0.05). The level of NF-*κ*B protein was significantly decreased in the LVD group compared to the LV group. The level of NF-*κ*B protein in the LLVD group was decreased compared to the LLV group (*P* < 0.05) (Figure 5).

### 3.3. Desflurane Attenuates Lung Injury in VILI

**Figure 6:**
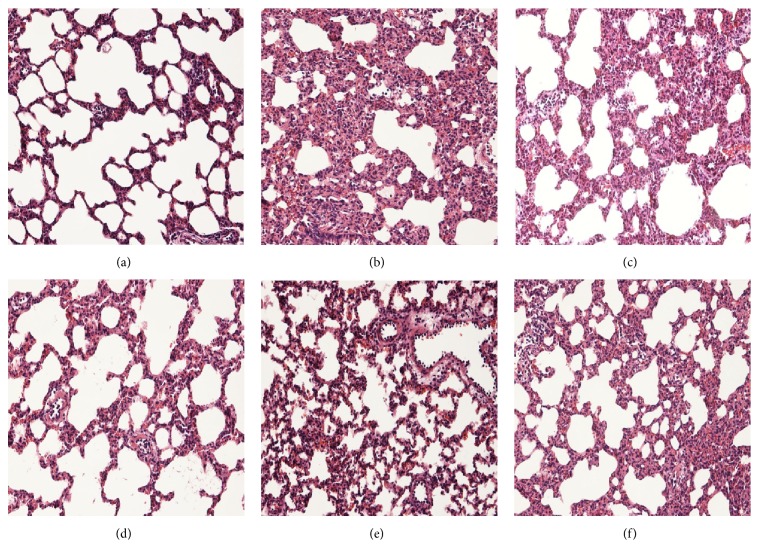
Histopathological analysis of lung tissues in the S group (a); C group (b); LV group (c); LVD group (d); LLV group (e); and LLVD group (f). (a)–(f) ×200. The lungs in rats in the LV and LVD groups showed a thickened alveolar wall, edema, less alveolar space, and obvious inflammatory cell infiltration. Similar to the LV and LVD groups, histological injury in the LLVD group was significantly ameliorated compared to the LLV group.

In rats in the LV group, light microscopic evaluation of the right lung revealed typical VILI pathophysiology, including lung mesenchymal edema, severe thickening of the alveolar wall, red blood cell leakage, and infiltration of macrophages and neutrophils in lung parenchyma. In rats in the LVD group, these visible signs of VILI were obviously attenuated (Figure 6). Histological score in the C group (2.8 ± 0.6), LV group (3.3 ± 0.4), and LVD group (2.5 ± 0.5) was significantly higher than in the S group (1.1 ± 0.3), and histological score in the LVD group was significantly lower than in the LV group (*P* < 0.05). Histological score in the LLVD was significantly lower than in the LLV group (Figure 6).

## 4. Discussion

The application of desflurane in patients with lung injury or lung dysfunction is debatable because of some reported negative effects on airway mechanics and microvascular permeability [16–18]. In contrast, other studies demonstrated that desflurane has a protective effect on lung function [11–15]. Therefore, this study was conducted to evaluate the effect of desflurane on lung injury. The results show that desflurane ameliorated VILI in a rat model of ARDS. Desflurane reduced histopathological changes of VILI, as well as local and systematic inflammation compared to oxygen-treated rats. Furthermore, desflurane increased survival in mechanically ventilated ARDS rats.

The use of systemic administration of LPS to induce pathological changes in rat lungs provides a simple and highly reproducible method for evaluating mechanisms of sepsis induced ARDS. In contrast, intravenous administration of LPS affects host inflammatory responses and does not simulate the lung injury found in patients undergoing lengthy surgeries. Severe ARDS may also be induced by gastric acid aspiration, which causes local lung injury in areas that come into contact with the acid. Notably, approximately 50% of the ARDS encountered in the clinic is induced by sepsis resulting from a Gram-negative infection, and another 25% is caused by aspiration [21]. Therefore, in this study we administrated LPS to develop a rat model of ARDS.

The pathophysiology of ARDS and VILI includes activation of inflammatory cells, release of proinflammatory factors, impairment of alveolar-capillary permeability, decrease in compliance, lung edema, severe hypoxemia, and lung injury [7, 8, 22]. Cytokines play a key role in VILI, causing local and systematic effects that can contribute to multiple organ failure [23, 24]. Evidence suggest that volatile anesthetics may ameliorate lung injury [9, 10, 12, 13, 25–27]; however, to the best of our knowledge, there are no published studies investigating the effect of desflurane on VILI. Furthermore, most studies investigating the influence of volatile anesthetics on VILI were performed using healthy lungs [9, 10]. However, in clinical practice, many patients that have prior lung injury require mechanical ventilation. These patients are most susceptible to VILI. Therefore, in this study, we investigated the effect of desflurane on VILI in an ARDS model. These data suggest that desflurane is a suitable anesthetic for patients susceptible to VILI. To mimic the course of human disease, we induced ARDS in rats with a sublethal dose of lipopolysaccharide [19].

In this study, we found that the desflurane significantly increased PaO_2_ and decreased the wet/dry ratio and protein content in BALF after administration of lipopolysaccharide and mechanical ventilation. Desflurane also reduced the total cell count, macrophages, neutrophils, and elastase levels in BALF. These results suggested that desflurane reduced the local inflammatory response caused by lipopolysaccharide and mechanical ventilation and improved the permeability of the alveolar-capillary membrane and gas exchange.

IL-8 is an important adhesive molecule for macrophages; blockage of IL-8 can ameliorate lung injury [28, 29]. IL-1*β*, TNF-*α*, and neutrophil elastase are proinflammatory factors that aggravate the inflammatory response and directly injure lung tissue in VILI [30]. NF-*κ*B regulates the inflammatory response by altering the production of the pro- and anti-inflammatory factors [31]. Blockage of the NF-*κ*B pathway can attenuate acute lung injury in isolated perfused lungs obtained from BALB/C mice [32]. Inhibition of NF-*κ*B can reduce the production of chemotactic factors in endothelial, epithelial, and resident macrophages in mouse lung [33, 34]. Therefore, we speculated that desflurane may influence the production of proinflammatory cytokines through an NF-*κ*B-mediated pathway [35, 36]. In the current study, the levels of NF-*κ*B protein were significantly increased after lipopolysaccharide administration and mechanical ventilation but significantly decreased by desflurane. IL-10 is also affected by desflurane [12, 36]. As an anti-inflammatory factor, IL-10 can antagonize the effects of proinflammatory factors and inhibit inflammatory cell migration [37].

Inflammation induced by ARDS and VILI may lead to a peripheral inflammatory response [19, 38]. In this study, rats receiving desflurane had decreased peripheral blood concentrations of TNF-*α*, IL-1*β*, and IL-8 compared to rats receiving oxygen. These data suggest that systemic inflammation was reduced by desflurane, possibly through the inhibition of NF-*κ*B and the local inflammatory response.

This study was associated with several limitations. First, we were unable to record airway pressure and compliance because the tidal volume in rats is too small. Desflurane may cause greater airway constriction than sevoflurane [18, 39], which may have a negative effect on the airway and have influenced the outcomes of this study. Second, mortality was high, possibly because of lung injury induced by a combination of LPS and VILI with a large tidal volume. Last, in this study, VILI was induced by a tidal volume of 30 ml/kg [20], which is substantially higher than the 8 ml/kg tidal volume that is recommended for critically ill patients or those at risk of ARDS [40, 41]. Our preliminary experiments revealed that a tidal volume of 10–15 ml/kg did not decrease the PaO_2_/FiO_2_ ratio in rats. Therefore, we used a higher tidal volume to mimic the PaO_2_/FiO_2_ ratio of the patients with prior lung injury and gas exchange dysfunction and the pathophysiological changes characteristic of VILI.

## 5. Conclusion

In conclusion, desflurane may represent a therapeutic modality for VILI in patients with prior lung injury. Desflurane may be a suitable anesthetic for these patients.
