# Corrigendum to “Desflurane Attenuates Ventilator-Induced Lung Injury in Rats with Acute Respiratory Distress Syndrome”

**DOI:** 10.1155/2021/9589313

**Published:** 2021-02-09

**Authors:** Xue Lin, Ying-nan Ju, Wei Gao, Dong-mei Li, Chang-chun Guo

**Affiliations:** ^1^Department of Anesthesiology, The Second Affiliated Hospital of Harbin Medical University, Harbin, Heilongjiang Province 150081, China; ^2^Department of Intensive Care Unit, Harbin Medical University Cancer Hospital, Harbin, Heilongjiang Province 150081, China

In the article titled “Desflurane Attenuates Ventilator-Induced Lung Injury in Rats with Acute Respiratory Distress Syndrome” [1], the authors have identified that the incorrect images were included in Figure 6 due to an error with the image selection during manuscript preparation. Figure 6 should be updated as follows:

The corresponding section in Section 3.3 “Desflurane Attenuates Lung Injury in VILI” should be corrected from:

Histological score in the C group (2.8 ± 0.6), LV group (3.3 ± 0.4), and LVD group (2.5 ± 0.5) was significantly higher than in the S group (1.1 ± 0.3), and histological score in the LVD group was significantly lower than in the LV group. Histological score in the LLVD was significantly lower than in the LLV group (Figure 6).

To:

Histological score in the C group (2.7 ± 0.4), LV group (3.2 ± 0.5), and LVD group (2.3 ± 0.5) was significantly higher than in the S group (1.1 ± 0.2), and histological score in the LVD group was significantly lower than in the LV group. Histological score in the LLVD was significantly lower than in the LLV group (Figure 6).

The authors confirm that this does not affect the conclusions of the article, and the corrigendum is being published with the agreement of editorial board.

## Figures and Tables

**Figure 1:**
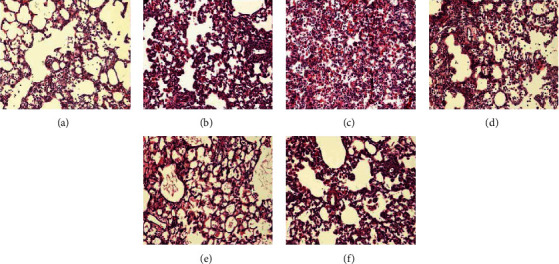
Histological score in the C group (2:7 ± 0:4), LV group (3:2 ± 0:5), and LVD group (2:3 ± 0:5) was significantly higher than in the S group (1:1 ± 0:2), and histological score in the LVD group was significantly lower than in the LV group. Histological score in the LLVD was significantly lower than in the LLV group (Figure 6).
